# Impact of Insoluble Dietary Fiber and CaCl_2_ on Structural Properties of Soybean Protein Isolate–Wheat Gluten Composite Gel

**DOI:** 10.3390/foods12091890

**Published:** 2023-05-04

**Authors:** Wentao Lian, Qinlin Hu, Min Qu, Bingyu Sun, Linlin Liu, Ying Zhu, Xiaoyu Xia, Yuyang Huang, Xiuqing Zhu

**Affiliations:** College of Food Engineering, Harbin University of Commerce, Harbin 150028, China; 18214853776@163.com (W.L.); huangyuyang1979@163.com (Y.H.)

**Keywords:** gelation, soybean protein isolate, soybean insoluble dietary fiber, CaCl_2_

## Abstract

The effect and mechanism of soybean insoluble dietary fiber (SIDF) (0~4%) and CaCl_2_ (0~0.005 M) on the properties of soybean protein isolate (SPI)–wheat gluten (WG) composite gel were studied. It was revealed that the addition of insoluble dietary fiber (1~2%) increased the strength and water-holding capacity (WHC) of the composite gel (*p* < 0.05) and enhanced the gel network structure compared with the control. WHC and LF-NMR showed that the water-binding ability of the gel system with only 2% SIDF was the strongest. The addition of excessive SIDF increased the distance between protein molecules, impeded the cross-linking of protein, and formed a three-dimensional network with low gel strength. The infrared spectrum and intermolecular force indicated that the interaction between SIDF and SPI were mainly physical, and the hydrophobic interaction and disulfide bond were the main forces in the gel system. The addition of CaCl_2_ can increase the critical content of gel texture destruction caused by SIDF, and the gel strength attained its peak at 3% SIDF, indicating that appropriate CaCl_2_ improved gel structure weakening caused by excessive SIDF. This study provides insights in enhancing the production of multi-component composite gel systems.

## 1. Introduction

Protein gels possess three-dimensional network structures formed by chemical forces between denatured protein molecules [1]. SPI is often used as the main raw material in gel production due to its high protein level and good gelling properties [2,3,4]. However, the application and development of natural soy protein gel products are greatly affected by their loose structure and high expansion rate. Wheat gluten, on the other hand, possesses the gel properties of proteins, and on interaction with SPI, inhibits the structural refolding of the protein and maintains the charged state on the protein surface. This leads to protein composite gels with high solubility [5]. Meanwhile, this is due to the mechanical interaction between SPI and WG. SPI is captured by WG’s continuous protein network in order to inhibit the swelling behavior of SPI gel during preparation. This preserves the original form of SPI gel [6], making it more suitable for food processing applications. Protein gel is an elastic gel formed by the network structure of protein molecules that are crosslinked with each other [3]. SPI and WG form hydrogen bonds with water through hydrophilic and polar groups to absorb water to the surface or interior of the structure during gel molding. The swelling and adhesion behaviors occur over time, while the internal water state of the system is constantly adjusted to form a continuous network structure [7]. The presence state and distribution of water molecules in protein gel will change, which leads to water migration after different processing [8]. Therefore, water migration is closely related to the structure of the protein gel network and affects the gel properties of protein gel. A multi-component composite gel system is a kind of material composed of two or more monomers formed by a crosslinking mechanism. Its wide use is closely related to its flexibility and controllability. It is widely used in medical instruments, cosmetics, adhesives, food processing, and other practical and industrial fields.

The larger specific surface area of SIDF fibers leads to the exposure of more hydrophilic groups, which leads to the excellent water absorption of SIDF, and the three-dimensional structure of SIDF also amplifies its adsorption [9]. SIDF also has excellent water- and oil-holding properties, in vitro antioxidant activity, and low price, which improves its edible value. Moreover, it has been implicated in reducing the risk of intestinal disease, cardiovascular disease, and diabetes [10,11,12]. Studies on protein–polysaccharide composite gels have revealed that these composite gels elicit better functional performance and stability than the single component gel [13,14]. The interactions are generally classified into two types [15]: one is covalent binding between proteins and polysaccharides through the Maillard reaction; the other is non-covalent binding through electrostatic attraction [16], hydrophobic interactions, and hydrogen bonds. Fang et al. [17] combined SPI and soybean soluble dietary fiber (SSDF) to study the gel performance of the new double-cross-linked gel, and found that the addition of SSDF significantly improved the energy storage modulus value and gel strength of SPI gel. Zhou et al. [18] studied the interaction between wheat dietary fiber and wheat gluten and found that the degree of interaction was mainly determined by the expansion degree and hydration degree of SIDF. However, this current research is limited to the synthesis of single-component proteins and polysaccharides: dietary fiber–sturgeon protein complex gel [19] and SIDF–potato protein gel [20]; however, there are few studies on the multi-component composite gel systems.

Salt ions play an important role in gel properties. The presence of salt ions does not only induce the exposure of internal protein hydrophobic groups [21], but also reduces the electrostatic repulsion between protein molecules through the electrostatic shielding effect [22], thereby promoting cross-linking and aggregation of protein molecules. Zhou et al. [21] found that addition of sodium ions with concentrations of 10~20 mM enhanced the hardness, WHC, and thermal stability of the gel. The Ca^2+^ and Cl^-^ in CaCl_2_ are attractive to water molecules. Wang et al. [23] also found that calcium ions can act as bridges between anionic groups of two protein molecules, linking them together with a “salt bridge” to enhance cross-linking and aggregation between proteins. However, Zhang et al. [24] found that the presence of calcium ions competed for water in the system, increased the irregular aggregation behavior of proteins, and reduced the WHC and gel strength. In summary, SIDF can absorb water, expand to fill the mesh structure, and improve the gel strength, while CaCl_2_ can change the water attraction effect to promote the formation and stability of the gel. These studies are limited to the single use of salt ions and fiber. Additionally, there are few studies on whether calcium ions and SIDF have synergistic effects on the gelation behavior of composite protein gel.

In this study, the composite gel was prepared by heat induction, and its gel properties and mechanical properties were studied by a rheological test and a texture profile analysis (TPA). Low-field NMR was used to interpret the water distribution of the composite gel. The molecular interaction and Fourier infrared spectroscopy were used to investigate the protein structure of the composite gel. Finally, the micromorphology of the composite gel was analyzed by scanning with an electron microscope. Studying the effects of this combination we can understand how to improve the nutritional value and quality of food products by adjusting the formula, and also has important implications for the development of novel foods.

## 2. Materials and Methods

### 2.1. Material

SPI (91.9% protein content) was purchased from Harbin Xichi Biotechnology Co., Ltd. (Harbin, China). WG (85.1% protein content) was purchased from Shandong Qufeng Food Technology Co., Ltd. (Weifang, China). SIDF (89.2% total dietary fiber content, 2.3% protein content) was purchased from Shaanxi Chenming Biotechnology Co., Ltd. (Xi’an, China) with purity ≥ 90%. Other reagents used were of analytical grade and purchased from Tianjin Reference Chemical Reagent Co., Ltd. (Tianjin, China).

### 2.2. Gel Preparation

SPI and WG (SPI:WG = 7:3) were dispersed in deionized water and stirred with a magnetic agitator (600 rpm) at 25 °C for 60 min to obtain an SPI-WG (SPI and WG complex) composite solution with a protein addition of 16% (*w*/*w*). SIDF (0~4%) and CaCl_2_ (0~0.005 M) were added to the SPI-WG composite solution and stirred with a magnetic agitator (DF-101S, LICHEN, Shanghai, China) (800 rpm) at 25 °C for 60 min to ensure a uniform distribution of components. Sodium azide (0.1%) was added to prevent deterioration, and the sealed samples were placed in a 4 °C refrigerator (BCD-510WDEM, Haier, Qingdao, China) for 12 h to ensure complete hydration. The sample solution was placed in a sealed device and heated in a 95 °C water bath for 30 min. The resulting gel was immediately cooled and stored in a 4 °C refrigerator for 12 h for later use.

### 2.3. Water-Holding Capacity

The WHC of the gel was performed in accordance with the centrifugation method described by Zhao et al. [25] with slight modifications. The 2 g (*M*1) gel samples were centrifuged (TG16-WS, BIORIDGE, Shanghai, China) at 10,000 rpm for 20 min. The surface water of the gel samples was blotted dry with filter paper and weighed. The mass of the gel sample after centrifugation was recorded to *M*2, and the *WHC* of gel was calculated according to the following formula.

$$WHC(\%)=\frac{M2}{M1}\times 100\%$$

### 2.4. Texture Profile Analysis

The samples were subjected to TPA analysis, and the gelation properties (elasticity and hardness, etc.) were determined using a texture analyzer (TA-XT Plus C, SMS, London, UK). Each sample was cut into 2 cm × 2 cm × 2 cm cubes. Typical parameters are as follows: pre-test speed 1.0 mm/s; test speed 5.0 mm/s; post-test speed 5.0 mm/s and trigger force 5 g. The compression ratio was 40% of the gel sample’s height. Each sample was analyzed 3 times.

### 2.5. Rheology

The dynamic rheology of the composite gel was measured by a rheometer (MCR102, Anton Paar, Shanghai, China) in accordance with the method of Zhuang et al. [26] with slight modifications. The gel sample was loaded onto the parallel plate of the rheometer with a diameter of 60 mm and the fixed spacing was 1 mm. A thin layer of silicone oil was applied to the protection seal to prevent the evaporation of water, and the lid was used to limit evaporation and mitigate interference (external noise and temperature). Samples were equilibrated on a parallel plate at 25 °C for 2 min before each measurement. The frequency scanning test was carried out in the range of 0.1–10 Hz, the strain was 0.5%, and 13 data points were measured. The viscoelasticity of the composite gel was determined by the storage modulus (G′) and loss modulus (G″).

### 2.6. Low Field Nuclear Magnetic Resonance (LF-NMR)

The composite gels were subjected to LF-NMR in accordance with the method of Schreuders et al. [27] with slight modifications. The gel sample was placed in a 7 mm MRI tube, which was covered to prevent water loss. The Benchtop NMR analyser (PQ001, NiuMai, Changchun, China) was used for analysis. The sampling frequency was 100 kHz, the pulse width of 90° was 18 μs, the number of echoes was 6000, and the interval time of repeated sampling was 10 s. The same sample was collected 8 times. Each sample was measured in parallel 3 times. The LF-NMR analysis software was used to collect the data and perform the inversion. The SIRT method was selected for the inversion. The data selection method was sampling, with the number of data being 500, filtering tap 3, relaxation time 100, minimum relaxation time 0.01 ms, maximum relaxation time 10,000 ms, and iteration times 10,000.

### 2.7. Molecular Force Measurement

The molecular force measurement was conducted in accordance with the method of Wang et al. [23] with modifications using Extraction solvent: S1: 0.6 mol/L NaCl; S2: 0.6 mol/L NaCl + 1.5 mol/L urea; S3: 0.6 mol/L NaCl + 8 mol/L urea; S4: 0.6 mol/L NaCl + 8 mol/L urea + 1.5 mol/L β-mercaptoethanol.

Sample extraction and dilution: Gel samples were freeze-dried and ground through an 80-mesh sieve. The powdered sample (0.25 g) was put into a 50 mL centrifuge tube; 5 mL of the four abovementioned solvents were added and mixed evenly. The solution was extracted at 4 °C for 5 h, centrifuged (TG16-WS, BIORIDGE, Shanghai, China) (10,000 rpm, 10 min) and the supernatant was stored at 4 °C for later use. The abovementioned four solvents (5 mL each) were added to the precipitation again, and the extraction was repeated twice. The protein content in the supernatant was determined by Coomassie brilliant blue method in the spectrophotometer (UV-1100, MAPADA, Shanghai, China) (determination wavelength 595 nm). Chemical bond representation method (protein content): Ionic bond: S1; hydrogen bond: S2–S1; hydrophobic interaction: S3–S2; and disulfide bond: S4–S3.

### 2.8. Fourier Transform Infrared Spectrum (FTIR)

FTIR analysis was carried out according to the method of Wang et al. [28] with appropriate modifications; the spectral data were recorded on the Fourier Infrared Spectrometer (PerkinElmer, Waltham, MA, USA). P_2_O_5_ was used to thoroughly freeze-dry powder in a dryer. The sample (2 mg) was accurately weighed and directly placed on a germanium crystal surface of a horizontal ATR instrument. The sample plate required for the FTIR experiment was obtained after tablet pressing. A total of 32 scans were carried out, the absorption spectrum was measured in the band range of 4000~400 cm^−1^, and the background spectral scanning resolution was 4 cm^−1^. The scanning time of each sample was about 1 min, and three parallel tests were conducted. Spectrogram analysis was performed using Peak Fit v4.12 software to assess the secondary structural changes of the proteins.

### 2.9. Scanning Electron Microscope (SEM)

The gel samples were fixed with 2.5% glutaraldehyde, cleaned with phosphate buffer (0.1 mol/L KH_2_PO_4_, pH 7.0), dehydrated with ethanol of different gradients, and then freeze-dried. The freeze-dried samples were coated with gold and observed using an SEM (Zeiss Supra 55, Zeiss, Germany) equipped with a voltage of 15 kV and a magnification of 2000 times.

### 2.10. Statistical Analysis

Data were subjected to ANOVA and Tukey’s test (*p* < 0.05) using Statistical package for social sciences (SPSS, version 20) software. Graphs were plotted using Origin 2021 software. All of the tests were performed in triplicate under identical conditions, and data were presented as mean ± standard deviation.

## 3. Results and Discussion

### 3.1. Effect of SIDF and CaCl_2_ on WHC of Composite Gel

**Figure 1:**
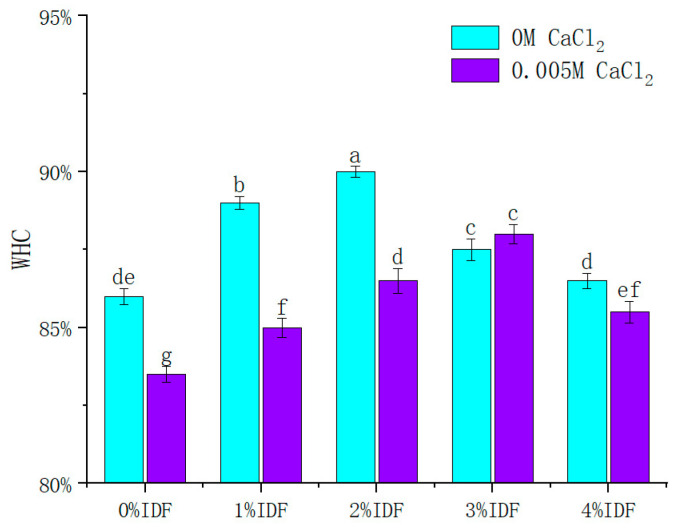
WHC of SPI-WG composite gel at different SIDF and CaCl_2_ concentrations. Different lowercase letters indicate significant differences between samples (*p* < 0.05).

Gel systems can be regarded as a network system filled with water molecules in a grid, and WHC is an important index to detect the change of water in the gel network caused by capillary effect [22]. The WHC changes in protein gels with different concentrations of SIDF and CaCl_2_ are shown in Figure 1. The addition of CaCl_2_ decreased the WHC of the protein gel, which may be because calcium ions competed with proteins or polysaccharides for water, thereby promoting the aggregation of proteins and polysaccharides and decreased their interaction with water molecules [24]. The WHC of all composite gel containing SIDF was significantly higher than that of the control (*p* < 0.05), indicating that the presence of SIDF can enhance the ability of the protein gel network to bind water [29]. The increase in WHC may be attributed to the formation of hydrogen bonds between the hydrophilic groups and the water molecules on the hydrophilic SIDF [25], as well as the construction of well-structured gel networks that favored the physical envelopment of water molecules. Water can be bound between protein networks or combined with functional groups of proteins in a gel system [30]. WHC essentially depends on the structural stability of the gel. The addition of excess fibers (3~4%) decreases the WHC of the gel, as shown in Figure 1, indicating that the presence of SIDF may damage the gel network and cause the loss of water. Sanchez et al. [31] also reported a similar phenomenon in the gel of surimi protein, where wheat dietary fiber had a dispersion effect in the gel system. The dispersion of fibers into proteins in the gel and the occurrence of hydrophobic contacts between proteins and fibers lead to impaired intermolecular protein cross-linking possibilities, the non-specific coagulation of surimi proteins, and the disruption of gel networks. For the 0 M CaCl_2_ group and 0.005 M CaCl_2_ group, it was found that the WHC of gel increased and then decreased with the increase in SIDF concentration (0~4%). However, 3% SIDF supplementation in the 0.005 M CaCl_2_ group was significantly higher than the 2% SIDF supplementation of the 0 M CaCl_2_ group at its WHC peak. These results suggested that there may be a synergistic effect between CaCl_2_ and SIDF, which promoted the addition of more SIDF without damaging the structure of the gel network and WHC.

### 3.2. Effect of SIDF and CaCl_2_ on Texture Properties of Composite Gels

Hardness is a mechanical textural property that describes the force required to deform or penetrate a product, and it is the internal bond that holds the food shape [30]. The texture analysis of the composite gel containing SIDF and CaCl_2_ is shown in Table 1. The addition of CaCl_2_ reduced the hardness of the composite gel. This may be because the addition of CaCl_2_ decreases electrostatic repulsion, enhancing the self-aggregation behavior of protein molecules [26], weakening the binding ability of protein and water molecules, forming an uneven gel network, and thus reducing its texture properties. For the composite gels without calcium ions, hardness and chewiness increased with the increase in SIDF (0~2%) content. This is because SIDF absorbed part of the water to improve the relative concentration of protein in the system and enhanced the degree of cross-linking between proteins. Chewiness showed a similar trend to hardness. However, at high SIDF content, the presence of CaCl_2_ greatly improved the gel chewiness. Meanwhile, the rigid filling effect of SIDF made up for the damage of non-gel components and even improved gel strength and WHC. The gel network is affected by the components and their interactions in the system. Wang et al. [23] also found that the addition of dietary fiber promoted the enhancement of hydrogen bonds and hydrophobic interaction between proteins and improved the texture properties of the gel. However, high concentrations of SIDF (3~4%) were over-dispersed in the gel network, which may hinder the cross-linking behavior between the proteins and cause irregular aggregation, thereby reducing the gel strength.

Springiness represents a gel’s ability to return to its original shape after deformation [32], which is shown in Table 1. There was no significant difference in the elastic values of the composite gels containing both SIDF and CaCl_2_, which may be due to decrease in the elastic fluctuation range of the high-concentration protein gel under puncture force. Cohesiveness represents the state or degree of gel aggregation. Cohesiveness reached its maximum value at 0 M CaCl_2_ + 2% SIDF, followed by 0.005 M CaCl_2_ + 3% SIDF, indicating that the addition of SIDF can increase the cohesiveness of the composite gel, whereas increasing SIDF concentration (4%) decreased gel textural properties by creating more heterogeneous and unstable network structures [33]. Meanwhile, the original structure of calcium-induced gel gradually formed a high-density structure due to the addition of SIDF, which was consistent with the results of Fang et al. [17], and also explained the reason why the peak value of the textural properties of 0.005 M CaCl_2_ shifted to the right.

Overall, the addition of the appropriate content of SIDF improved gel textural properties, and the existence of CaCl_2_ may prolong the critical point of SIDF failure, which promoted the addition of more SIDF.

### 3.3. Effect of SIDF and CaCl_2_ on Rheology Properties of Composite Gel

**Figure 2:**
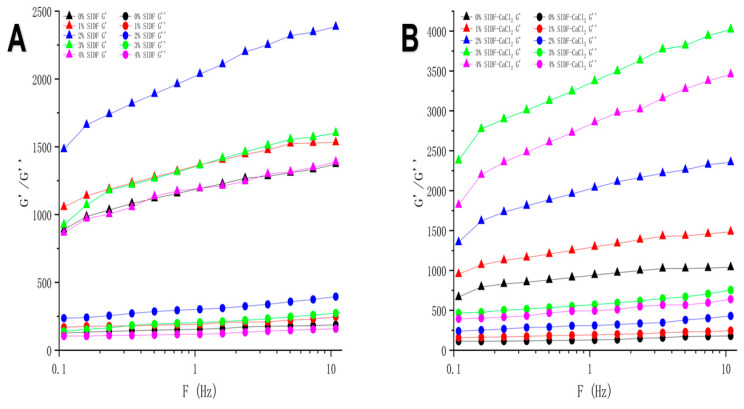
Rheology of SPI-WG composite gel at different SIDF and CaCl_2_ concentrations: (**A**) 0 M CaCl_2_ G′ and G″; (**B**) 0.005 M CaCl_2_ G′ and G″.

The influence of SIDF and calcium ions at different concentrations on the elasticity of the composite gel is shown in Figure 2. The G′ value of all gel samples was always greater than the G″ value without crossover, which confirmed that all gel samples had elastic and gelation behaviors [34].

The storage modulus (G′) and loss modulus (G″) values of the composite gels showed different patterns under different salt ion concentrations. The electrostatic shielding effect caused by calcium ions led to a stronger interaction between proteins, speeding up the rate of protein polymerization into protein gel and the formation of an uneven coarse gel network [23]. Therefore, the uneven gel network caused by the addition of CaCl_2_ corresponds to the decrease in G ‘value. The addition of SIDF had a significant effect on the solid viscoelasticity of the composite gel. The G′ and G″ of the composite gel increased with the increase in SIDF concentration from 0~2%, and decreased with the increase in SIDF concentration from 3~4%, as shown in Figure 2A. This is the water absorption of SIDF in the process of thermogenic gel formation. SIDF gathers water around itself, which inversely increases the relative concentration of local proteins and enhances protein interaction when low concentration of SIDF was added. The dense gel structure can effectively decrease the water molecules in the protein network and increase the G′ and G″ values of the gel system [35]. However, for high concentrations of SIDF, it was hypothesized that a large number of SIDF molecules was distributed in the gel system, which increased the water content around it and increased the distance between proteins. This would lead to a low degree of crosslinking between proteins and reduction in hydrogen and disulfide bonds, thereby weakening the gel performance of the gel system. Meanwhile, the structure of the dense gel network was weakened, and irregular voids were formed due to the increase in free water content at 3~4% SIDF concentration. The G′ and G″ of the compound gel showed a decreasing trend. Figure 2B also showed the G′ and G″ of the composite gel increasing at low SIDF concentration (0~3%) and decreasing at high SIDF concentration (4%).

The results showed that the solid viscoelastic performance of the composite gel was poor at high SIDF concentration. The presence of CaCl_2_ increased the G′ of the composite gel with high SIDF concentration compared to the control group, which was consistent with the results of gel texture.

### 3.4. Influence of SIDF and CaCl_2_ on Water Distribution of Composite Gel

**Figure 3:**
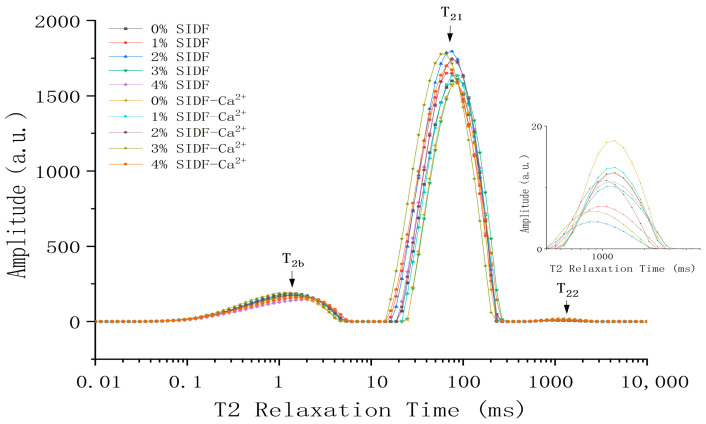
Changes in T_2_ relaxation time of SPI-WG composite gel at different SIDF and CaCl_2_ concentrations.

The faster the relaxation time, the more difficult it is for water molecules to move [36]. Three peaks, T_2b_, T_21_, and T_22_, appeared in the T_2_ relaxation time within 0.01 to 10,000 ms, as shown in Figure 3 T_2_ relaxation time is related to the mobility of water molecules in the system. T_2b_ represents combined water, that is, bonded with polar groups of large molecules, such as proteins, in the way of hydrogen bonding. T_21_ represents the water bound in the network structure of the gel, which is the most important water in the gel, and its mobility is related to the interaction of proteins or other components, also known as non-mobile water [37]. T_22_ represents the water that can move freely in the gel network system, also known as free water.

The gel sample group without any substance addition (0% SIDF) had the highest PT_22_ value, indicating that the pure protein gel contained abundant free water in the protein interior and interstitials. More free water (T_22_) was detected in the gel with CaCl_2_ compared to the gel without CaCl_2_, as can be seen in Figure 3 and Table 2. This may be because the enhanced protein–protein interaction weakened the protein–water interaction, leading to a large overflow of water, which is consistent with the results of water retention. In the whole gel system, the non-mobile water represented by T_21_ accounts for the largest proportion. This was because proteins undergo denaturation under heating conditions, and the expanded protein molecular chains were cross-linked and aggregated again, forming a three-dimensional network structure and binding water in the gel network [38]. There are differences of form and distribution of water under different dosages of SIDF in protein gel. The addition of SIDF reduces the relative content of PT_22_, and T_22_ moves towards a shorter relaxation direction. The shorter the transverse relaxation time, the shorter the spin recovery time of the hydrogen proton in the water molecule, the lower the degree of freedom [36]. In this state, the higher the degree of binding between water molecules and proteins, the stronger the binding force of the protein network to water. This accelerates the conversion of free water to non-mobile water in the gel matrix. This may be due to the physical capture of water by the porous surface of the SIDF [39]. Pereira et al. [35] also found that the addition of coconut fiber could shorten the T_2_ relaxation time of the gel when studying the effect of coconut fiber on myofibrillar gel. At the same time, an increase in protein relative concentration increased the ordered aggregation of proteins, and a large number of water-binding sites on protein side chains can better bind water in the gel network system [40], thereby improving its water-holding capacity and gel strength.

SIDF diffused between the gels, the hydrophilic groups facilitated more hydrogen proton binding, and the hydration of the gel matrix was accelerated [14]. However, T_22_ moves towards a longer relaxation direction, which indicates that the hydrogen proton is weakly bound and the water molecules are prone to phase transfer and flow when the amount of SIDF was 3~4%. SIDF has a dispersion effect in the gel network and absorbs a large amount of water. This increases the distance between protein molecules, creating a barrier effect of protein crosslinking. The protein gap was enlarged, and the part of T_21_ with high fluidity was discharged and converted into free water T_22_, which revealed the reason for the increase in water in the T_22_ group [8]. Meanwhile, the water-binding sites of the side chains of proteins decreased, which weakened cross-linking between proteins. More proteins aggregated in a disorderly state, weakening the structure of the gel network, and more water emerged as free water. This was consistent with the deterioration of the texture of the gel. CaCl_2_ can promote the aggregation behavior of proteins and fibers. Salt bridges can be formed to close the distance between proteins, prolong the critical point of SIDF destruction, and decrease the degradation of gel texture caused by excessive fiber. The above results confirmed that there was indeed a migration phenomenon between non-mobile water and free water in the gel system during the formation of protein gel, and it was greatly related to the texture of protein gel.

### 3.5. Analysis of the Interaction Force between Composite Gels

The intermolecular interaction forces of the composite gel are shown in Table 3. The order of the proportion of each bond is as follows: disulfide bond > hydrophobic interaction > ionic bond > hydrogen bond. The results show that the main forces to maintain the gel conformation were disulfide bond and hydrophobic interaction, and the secondary forces were ionic bond and hydrogen bond.

The low hydrophobic interaction may be due to the low water content and dense microstructure. The content of protein covalent aggregates increased, thereby reducing the movement of hydrophobic groups, leading to the formation of large insoluble aggregates in the gelation process with increase in disulfide bonds. With the addition of CaCl_2_, the ratio of ionic and hydrogen bonds decreased significantly, i.e., CaCl_2_ broke hydrogen bonds and electrostatic force. The transformation of the disulfide bond conformation is essential for the gel structure of protein [41]. The proportion of disulfide bonds in the gel with CaCl_2_ was higher than that in the gel without CaCl_2_. This was due to reduced electrostatic repulsion, which caused the proteins to be closer together and more sulfhydryl groups to be converted into disulfide bonds [23]. At low SIDF concentration, the addition of SIDF can absorb excess water and improve the relative concentration of protein in the gel system [42]. The increase in disulfide bonds and hydrophobic interactions indicated that the degree of cross-linking between protein molecules was increased and an ordered gel network was formed, which was consistent with the results of Jiang et al. [43]. A large amount of SIDF was distributed in the gel network with the increase in SIDF concentration, which inhibited protein aggregation and cross-linking behavior, deceased disulfide bond and hydrophobic interaction, and affected protein gelation. However, CaCl_2_ can continue to maintain a good degree of crosslinking through salt bridges, which further supports the textural and elastic properties results. Hydrophobic interaction is the main intermolecular force in gels. At the same SIDF concentration, the proportion of hydrophobic action in gels with CaCl_2_ addition was consistently higher than that without CaCl_2_ addition. On one hand, the reason is that calcium ions can combine with the negatively charged groups on the surface of protein and polysaccharide molecules, causing conformation changes and improving the interaction between protein molecules. On the other hand, the presence of CaCl_2_ may increase protein solubility, promote the exposure of hidden groups in proteins, and form a large number of hydrophobic groups [23].

The results show that the disulfide bond and hydrophobic interaction are the main forces in the composite gel system. The interaction in the gel system was enhanced at low SIDF concentration and weakened at high SIDF concentration. The presence of CaCl_2_ improved the degree of hydrophobic interaction in the system and slowed down the weakening degree of gel texture caused by high SIDF concentration.

### 3.6. Effects of SIDF and CaCl_2_ on Secondary Structure of Proteins

FTIR is a common method to determine the secondary structure of proteins [44], and the amide I band is usually used to analyze the content of the secondary structure of proteins [45,46]. The amide I band is mainly caused by a C-O stretching vibration (about 80%), some C-H stretching, and in-plane N-H bending, while the C-O in the protein molecule is mainly determined by its secondary structure changes. Reflecting it on the infrared spectrum, the β-sheet is located in 1600~1640 cm^−1^ band, the random coil is located in 1640~1650 cm^−1^ band, the α-helix is located in 1650~1660 cm^−1^ band, and the β-turn is located in 1660~1700 cm^−1^ band.

**Figure 4:**
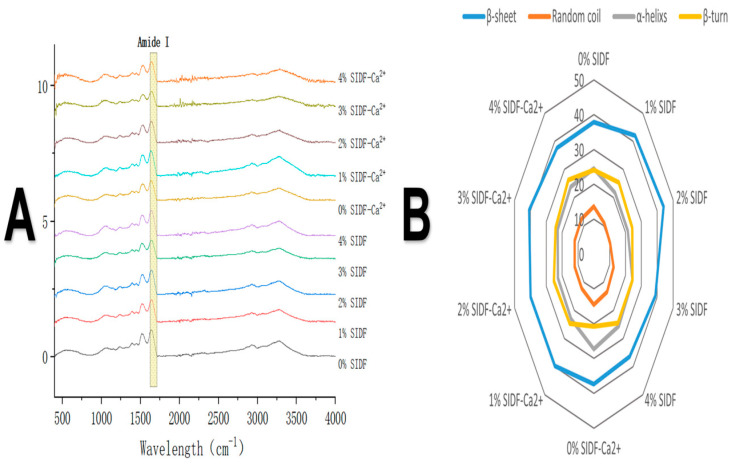
(**A**) infrared spectra and (**B**) secondary structure relative content of SPI-WG composite gel at different SIDF and CaCl_2_ concentrations.

Similar infrared spectra were found in all gel samples, and the addition of SIDF did not induce new peaks, as shown in Figure 4, indicating that no chemical reaction occurred between protein and SIDF, which was consistent with the results of Xiao et al. [22]. Some physical interactions between components were thought to be the main driving force between the cellulose chain and the protein molecule [47].

It was observed in Figure 4 that the addition of calcium ions promoted protein irregular aggregation, the content of α-helix in the system was more than that in the control group, and the relative content of β structure was decreased. The amide I band shifted to higher frequencies with the increase in SIDF concentration, indicating that the addition of SIDF promoted the expansion of the protein structure and enhanced intermolecular interactions, leading to the reduction in α-helix, which shifted the infrared spectra to higher frequencies. It was found that the addition of sugarcane insoluble dietary fiber to myofibrillar protein resulted in the left shift and right shift of the characteristic peaks of the gelatinized amide I and amide III bands, respectively, resulting in the transformation of α-helix to β-sheet and the formation of a compact and uniform gel network structure [14]. High SIDF concentration was irregularly aggregated and distributed in the gel system, which decreased the degree of protein cross-linking. The increase in the β structure ratio also indicated change in the ordered structure of proteins [48]. Overall, protein gels with better structural properties and network states have a low proportion of α-helix and a high proportion of β-sheet in the system.

### 3.7. Microstructure

**Figure 5:**
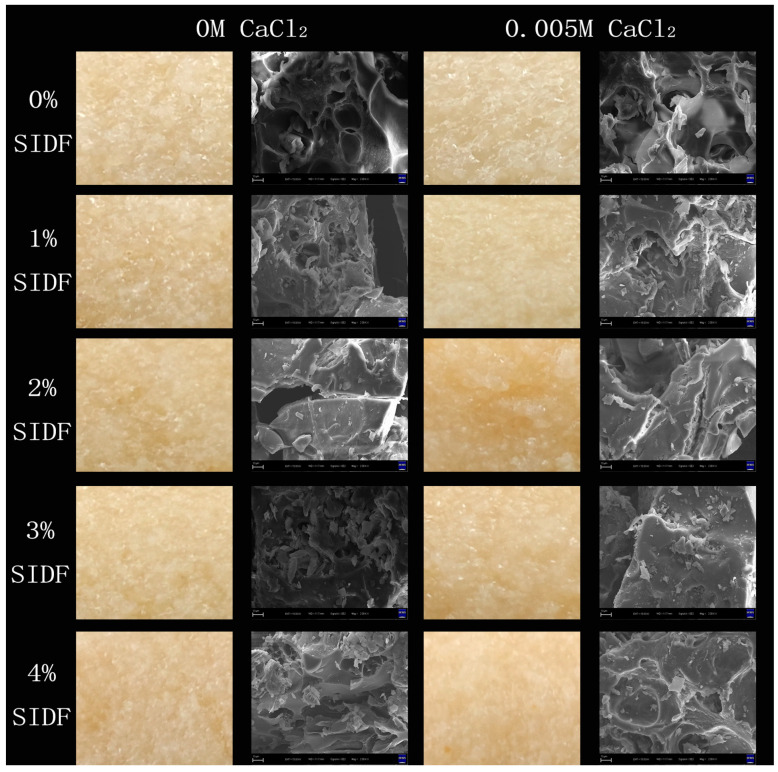
Physical images and SEM images of SPI-WG composite gel at different SIDF and CaCl_2_ concentrations (2000×).

The physical images and microstructures of the gels at different SIDF and CaCl_2_ concentrations are shown in Figure 5. SPI-WG composite gel had a rough network structure, and the pores in the system were large and uneven. These pores acted as water channels to weaken the integrity of the gel network during the gel-forming process. The addition of SIDF filled the gaps in the gel network at low SIDF concentration (1~2%), increased the gel hardness, and had a higher strength gel network [49]. Zhang et al. [50] also found that the addition of sodium carboxymethyl cellulose promoted the formation of a tight and ordered microstructure, and the dense structure gave the gel good texture properties and WHC. SIDF was excessively dispersed in the gel system at high concentrations of SIDF (3~4%), which increased the distance between protein molecules and hindered cross-linking between protein molecules. It was difficult to form an orderly protein gel network, and the structure was looser and the pores were enlarged. CaCl_2_ can continuously enhance the cross-linking between proteins by means of salt bridge to improve the gel structure weakening caused by excessive SIDF. However, protein molecules separated by SIDF were aggregated by CaCl_2_ with further increase in SIDF concentration. Meanwhile, large aggregators formed a gel network structure with a low crosslinking degree, and the gel had weak resistance to external forces [51]. The adjacent large aggregates in the gel slid under the action of external forces and weakened the network structure, thereby showing a low gel strength.

### 3.8. Formation Mechanism of SPI-WG Composite Gel

The seepage of water inside the gel forms “water channels” or water cavities, making the integrity of the gel network and the amount of gel decrease during the process of the conversion of protein solution to heat-induced gelation. SIDF can be used as a “filler” in the gel system to improve the texture of the gel. The presence of SIDF made the protein gel structure more dense, smooth, and ordered; the gel strength and WHC increased and the free water content decreased. This was attributed to fiber, which has good hydration ability, and part of the branch chains filled the pores of the gel network during the process of protein gelation. Meanwhile, the water in the water channel was transferred from the protein to the fiber, which effectively reduced the degree of water precipitation during the heating process and reduced the migration of water and the formation of the water channel. The SEM and low-field NMR results proved the above conclusions. Therefore, SIDF in appropriate concentrations can be used as a filler or proppant for promoting the integrity and densification of a gel structure.

A large number of hydrophilic groups on the SIDF molecular chain competed with proteins for water when the addition of SIDF was increased. The decrease in protein–water interaction was accompanied by an increase in protein–protein interaction and the formation of protein aggregates. At the same time, SIDF was dispersed in the gel system and blocked the gaps between the protein molecular chains, which reduced the conformational change of the protein. This allowed more hydrophobic regions to be well hidden within the protein structure, reducing the overall cross-linking and aggregation behavior between protein molecules. The structural properties were positively correlated with the relative contents of the β-sheet, β-turn, and random coil, and negatively correlated with the relative contents of the α-helix. The changes of protein secondary structure also indicated that the addition of excessive SIDF promoted the formation of scattered and uneven protein aggregates, resulting in the formation of a loose gel network structure.

**Figure 6:**
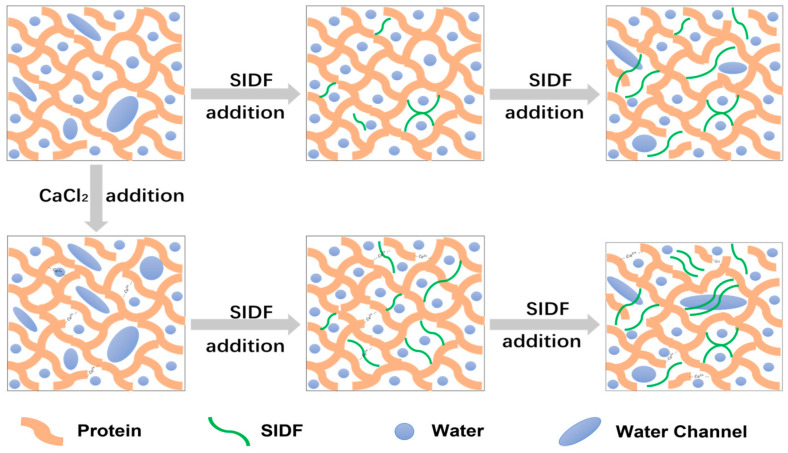
SPI-WG composite gel formation mechanism model under different SIDF and CaCl_2_ concentrations.

CaCl_2_ reduces electrostatic repulsion between and within proteins, resulting in more hydrophobic regions being exposed and a reduction in protein–water interactions. The over-aggregation of proteins plays a negative effect on composite gels. The strengthening of protein interaction caused by calcium ion electrostatic shielding can slightly delay the gel failure point when the gel failure critical point of SIDF is reached and promote the filling and addition of more SIDF while maintaining the gel state. However, with further addition of SIDF, the “aggregation power” of calcium ions is not enough to make up for the damage caused by SIDF, and the aggregation of proteins between SIDFs plays a greater role in the destruction of the gel network (Figure 6).

## 4. Conclusions

The effect of SIDF and CaCl_2_ on the structural and elastic properties of SPI-WG composite gel were studied. It was deduced that the integrity of the pure protein gel network was damaged by the large and uneven pores in the gel network. An appropriate amount of SIDF can fill the gel pores and enhance the network structure and gel properties. The protein gel with SIDF added had a denser three-dimensional structure and fewer holes in the gel, which improved the water-holding performance of the gel to a certain extent. However, the presence of excessive SIDF led to an overfilling effect, which hindered the cross-linking behavior between protein molecules and greatly reduced the gel strength. The appropriate amount of SIDF in different systems (with or without CaCl_2_) was different. In the absence of CaCl_2_, the maximum dosage of SIDF was 2%, which elicited the best gel performance. CaCl_2_ can delay the gel damage point and improve the gel structure weakening caused by excessive SIDF. However, the calcium salt bridge was insufficient to maintain the gel network as SIDF increased. The protein molecules further became scattered protein aggregates and eventually formed an uneven and discontinuous gel network. These results provide knowledge on the regulation of the quality of insoluble fiber–protein gel.

## Figures and Tables

**Table 1:** Textural properties of SPI-WG composite gel at different SIDF and CaCl_2_ concentrations. Different lowercase letters indicate significant differences between samples (*p* < 0.05).

Sample	0% SIDF	1% SIDF	2% SIDF	3% SIDF	4% SIDF	0% SIDF-CaCl_2_	1% SIDF-CaCl_2_	2% SIDF-CaCl_2_	3% SIDF-CaCl_2_	4% SIDF-CaCl_2_
Hardness (gf)	78.76 ± 0.24 i	108.71 ± 0.30 e	137.02 ± 0.41 a	130.49 ± 0.39 b	124.22 ± 0.37 c	65.44 ± 0.19 j	79.88 ± 0.24 h	85.65 ± 0.26 g	108.97 ± 0.34 d	97.44 ± 0.29 f
Springiness	0.98 ± 0.00 b	0.98 ± 0.01 b	0.97 ± 0.00 c	0.97 ± 0.01 c	0.98 ± 0.01 b	0.96 ± 0.00 d	0.99 ± 0.00 a	0.99 ± 0.02 a	0.99 ± 0.01 a	0.98 ± 0.00 b
Chewiness (gf)	36.34 ± 0.11 i	51.89 ± 0.16 f	72.53 ± 0.22 b	65.94 ± 0.19 d	57.27 ± 0.17 e	28.83 ± 0.09 j	39.15 ± 0.12 h	44.60 ± 0.13 g	82.09 ± 0.245 a	72.07 ± 0.22 c
Cohesiveness	0.47 ± 0.00 f	0.48 ± 0.01 e	0.54 ± 0.00 a	0.52 ± 0.00 b	0.47 ± 0.02 f	0.46 ± 0.01 g	0.49 ± 0.00 d	0.52 ± 0.01 b	0.54 ± 0.00 a	0.50 ± 0.00 c

**Table 2:** Relaxation time (T_22_) and percentage of relaxation area (P_22_) of SPI-WG composite gel at different concentrations of SIDF and CaCl_2._ Different lowercase letters indicate significant differences between samples (*p* < 0.05).

Sample	0% SIDF	1% SIDF	2% SIDF	3% SIDF	4% SIDF	0% SIDF-CaCl_2_	1% SIDF-CaCl_2_	2% SIDF-CaCl_2_	3% SIDF-CaCl_2_	4% SIDF-CaCl_2_
T_21_ (ms)	75.65 ± 0.00 c	65.79 ± 0.00 d	75.65 ± 0.00 c	86.97 ± 0.00 b	100.00 ± 0.00 a	86.97 ± 0.00 b	86.97 ± 0.00 b	75.65 ± 0.00 c	57.22 ± 0.00 e	65.79 ± 0.00 d
PT_21_ (%)	92.69 ± 0.22 fg	95.76 ± 0.14 c	97.71 ± 0.10 a	93.69 ± 0.41 de	93.29 ± 0.27 ef	89.20 ± 0.35 h	92.29 ± 0.21 g	94.37 ± 0.14 d	96.51 ± 0.17 b	93.08 ± 0.45 efg
T_22_ (ms)	1232.85 ± 0.00 b	932.61 ± 0.00 d	811.13 ± 0.00 e	1072.27 ± 0.00 c	1072.27 ± 0.00 c	1417.47 ± 0.00 a	1232.85 ± 0.00 b	1072.27 ± 0.00 c	811.13 ± 0.00 e	1232.85 ± 0.00 b
PT_22_ (%)	7.06 ± 0.23 bc	3.98 ± 0.12 f	2.05 ± 0.08 g	6.07 ± 0.27 de	6.49 ± 0.46 cd	10.59 ± 0.72 a	7.47 ± 0.32 b	5.39 ± 0.27 e	3.23 ± 0.16 f	6.67 ± 0.21 bcd

**Table 3:** Intermolecular interaction forces of SPI-WG composite gel at different SIDF and CaCl_2_ concentrations. Different lowercase letters indicate significant differences between samples (*p* < 0.05).

Sample	0% SIDF	1% SIDF	2% SIDF	3% SIDF	4% SIDF	0% SIDF-CaCl_2_	1% SIDF-CaCl_2_	2% SIDF-CaCl_2_	3% SIDF-CaCl_2_	4% SIDF-CaCl_2_
Ionic bond (mg/g)	11.87 ± 0.36 c	11.11 ± 0.44 cd	13.66 ± 0.39 b	15.18 ± 0.21 a	14.42 ± 0.43 ab	8.69 ± 0.26 e	7.29 ± 0.22 f	14.17 ± 0.42 b	11.49 ± 0.37 c	10.47 ± 0.31 d
Hydrogen bonds (mg/g)	7.29 ± 0.22 a	6.27 ± 0.18 b	5.89 ± 0.09 c	5.13 ± 0.13 c	2.84 ± 0.07 e	4.11 ± 0.16 d	7.04 ± 0.18 a	6.27 ± 0.21 b	4.37 ± 0.13 d	2.20 ± 0.09 f
Hydrophobic interactions (mg/g)	19.76 ± 0.59 e	23.84 ± 0.42 d	22.82 ± 0.68 d	20.27 ± 0.60 e	19.89 ± 0.37 e	24.47 ± 0.73 d	27.40 ± 0.82 c	34.41 ± 1.03 a	30.07 ± 0.77 b	30.58 ± 0.91 b
Disulfide bonds (mg/g)	39.49 ± 1.18 e	43.05 ± 1.31 cd	49.67 ± 0.96 a	46.62 ± 1.35 b	43.18 ± 1.28 cd	34.66 ± 1.94 f	38.47 ± 0.88 e	42.67 ± 1.26 cd	44.96 ± 1.43 bc	40.51 ± 0.77 de

## Data Availability

The data presented in this study are available on request from the corresponding author. The data are not publicly available due to restrictions eg privacy or ethical.
